# Artificial Intelligence in Stroke Rehabilitation: A 20‐Year Bibliometric Analysis of Digital Health Trends and Technologies

**DOI:** 10.1002/brb3.71451

**Published:** 2026-04-27

**Authors:** Yuhua Li, Weixi Liu, Xiaomei Yuan

**Affiliations:** ^1^ Department of Neurology Qingyang People's Hospital Qingyang Gansu China; ^2^ Department of Trauma Surgery Qingyang People's Hospital Qingyang Gansu China

**Keywords:** artificial intelligence, bibliometric analysis, digital health, rehabilitation, stroke

## Abstract

**Background:**

Stroke remains a leading cause of long‐term disability worldwide, and rehabilitation is essential for recovery. Although artificial intelligence (AI)‐related technologies have received growing attention in stroke rehabilitation, the knowledge structure and thematic evolution of this interdisciplinary field remain unclear.

**Objective:**

To conduct a bibliometric analysis of AI‐related research in stroke rehabilitation from 2005 to 2024 and map publication trends, major contributors, thematic clusters, and emerging topics.

**Methods:**

Relevant publications were retrieved from the Web of Science Core Collection (WoSCC), including SCI‐Expanded and SSCI, on November 30, 2024. Only English‐language articles and review articles published between January 1, 2005, and November 30, 2024 were included. A total of 3436 records were analyzed using CiteSpace 6.4.R1 Basic, GraphPad Prism 10.1.2, and biblioshiny in R. Analyses covered publication trends, collaboration networks, journal distribution, keyword co‐occurrence, clustering, and burst detection.

**Results:**

Publication output increased markedly over time, with the United States contributing the largest number of publications. The Swiss Federal Institutes of Technology Domain was among the leading institutions, and Rocco Salvatore Calabrò was among the most productive and highly cited authors. Core publication venues included the *Journal of NeuroEngineering and Rehabilitation* and *IEEE Transactions on Neural Systems and Rehabilitation Engineering*. The literature mainly focused on virtual reality, upper‐limb rehabilitation, rehabilitation robotics, machine learning, cognitive rehabilitation, and transcranial direct current stimulation. Recent burst terms, including machine learning, artificial intelligence, and deep learning, indicated growing attention to data‐driven rehabilitation approaches.

**Conclusions:**

AI‐related research in stroke rehabilitation has expanded substantially, with increasing emphasis on adaptive, data‐driven, and technology‐assisted approaches. This study provides a descriptive overview of the field's major trajectories, emerging gaps, and interdisciplinary directions, and may help inform future research and translational exploration.

## 1 Introduction

Stroke is a leading cause of long‐term disability and the second leading cause of death globally, characterized by sudden disruption of cerebral blood flow resulting in irreversible brain tissue damage (WHO 2022). With global population aging accelerating, the incidence of stroke, including both first‐ever and recurrent events, is projected to increase substantially by 2030 (Béjot et al. 2019). Poststroke sequelae such as impaired mobility, speech dysfunction, and cognitive deficits not only reduce quality of life but also impose considerable socioeconomic burdens on families and healthcare systems (Bersano and Gatti 2023; Ju et al. 2022; Mulhern 2023). In this context, effective rehabilitation is not merely supportive care. It is a critical component of long‐term functional recovery and social reintegration, with important implications for disability‐adjusted life years (DALYs) and healthcare costs.

Traditional rehabilitation approaches, including physical, occupational, and speech therapy, have demonstrated value in restoring basic functional abilities (Khan 2023; S. Li 2023). However, these interventions rely heavily on therapist expertise and often require in‐person, resource‐intensive delivery (Shahid et al. 2023). As a result, conventional rehabilitation faces persistent challenges. These include limited personalization, high demands on personnel and infrastructure, and unequal accessibility, particularly in low‐resource settings (McCarty and Shanahan 2021; Magaqa et al. 2021). Many stroke survivors therefore remain underserved. This highlights the need for more scalable, adaptive, and individualized rehabilitation strategies.

In recent years, artificial intelligence (AI)‐related technologies have attracted increasing research attention in stroke rehabilitation. Approaches such as machine learning, deep learning, virtual reality (VR), and robotic‐assisted therapy have been increasingly explored in relation to rehabilitation assessment, prediction, monitoring, and intervention design (Qiu et al. 2022; L. Zhang, Jia, et al. 2022; Scott et al. 2022; Georgiev et al. 2021). At the same time, these technology‐related directions differ substantially in methodological maturity, clinical applicability, and translational pathway (Murakami et al. 2023; Bai et al. 2022; Tang et al. 2024). Despite the rapid growth of this interdisciplinary literature, there is still a lack of systematic quantitative assessment of how the field has evolved over time. Its publication patterns, thematic structure, and emerging research directions therefore remain insufficiently characterized.

To address this gap, the present study conducts a bibliometric analysis of AI‐related research in stroke rehabilitation from 2005 to 2024. Previous bibliometric studies have shown that long‐term mapping can effectively reveal structural patterns, research hotspots, and evolving thematic trends (X. Li et al. 2024; Jiang et al. 2025). By systematically examining publication growth, major contributors, collaboration patterns, thematic clusters, and emerging topics, this study aims to provide a clearer overview of the knowledge structure of the field. In addition to mapping publication trends, it also seeks to identify underrepresented rehabilitation domains, characterize methodological heterogeneity across AI‐related approaches, and highlight potential translational and collaborative gaps in the current literature.

## 2 Methods

### 2.1 Data Sources and Search Strategy

The literature search was conducted in the Web of Science Core Collection (WoSCC) on November 30, 2024, using the Science Citation Index Expanded (SCI‐Expanded) and Social Sciences Citation Index (SSCI). WoSCC was selected as the primary data source because it provides standardized bibliographic records, cited‐reference indexing, and broad coverage across medicine, neuroscience, rehabilitation, engineering, and related interdisciplinary domains. In addition, WoSCC is widely used in bibliometric research because its structured citation data are highly compatible with knowledge mapping tools such as CiteSpace and Biblioshiny, thereby supporting reproducible co‐citation, co‐authorship, and keyword co‐occurrence analyses (M. Zhang, Wang, et al. 2022; Wang et al. 2024; Deng et al. 2023). Nevertheless, because AI in stroke rehabilitation is an inherently interdisciplinary field, restricting retrieval to a single database may underrepresent some engineering‐oriented studies, conference proceedings, and computer science publications that are more comprehensively indexed in other sources. This should be taken into account when interpreting the coverage and representativeness of the retrieved literature.

A topic search (TS) was performed, which in WoSCC covers the title, abstract, author keywords, and Keywords Plus. To improve retrieval transparency and reproducibility, the search strategy was structured around three concept blocks: stroke‐related terms, AI/digital rehabilitation technology‐related terms, and rehabilitation‐related terms. These three blocks were combined using the Boolean operator AND. The exact query strings used in the search are provided in Table S1.

The search period was restricted to publications from January 1, 2005 to November 30, 2024. Only English‐language records were included, and document types were limited to articles and review articles.

**FIGURE 1:**
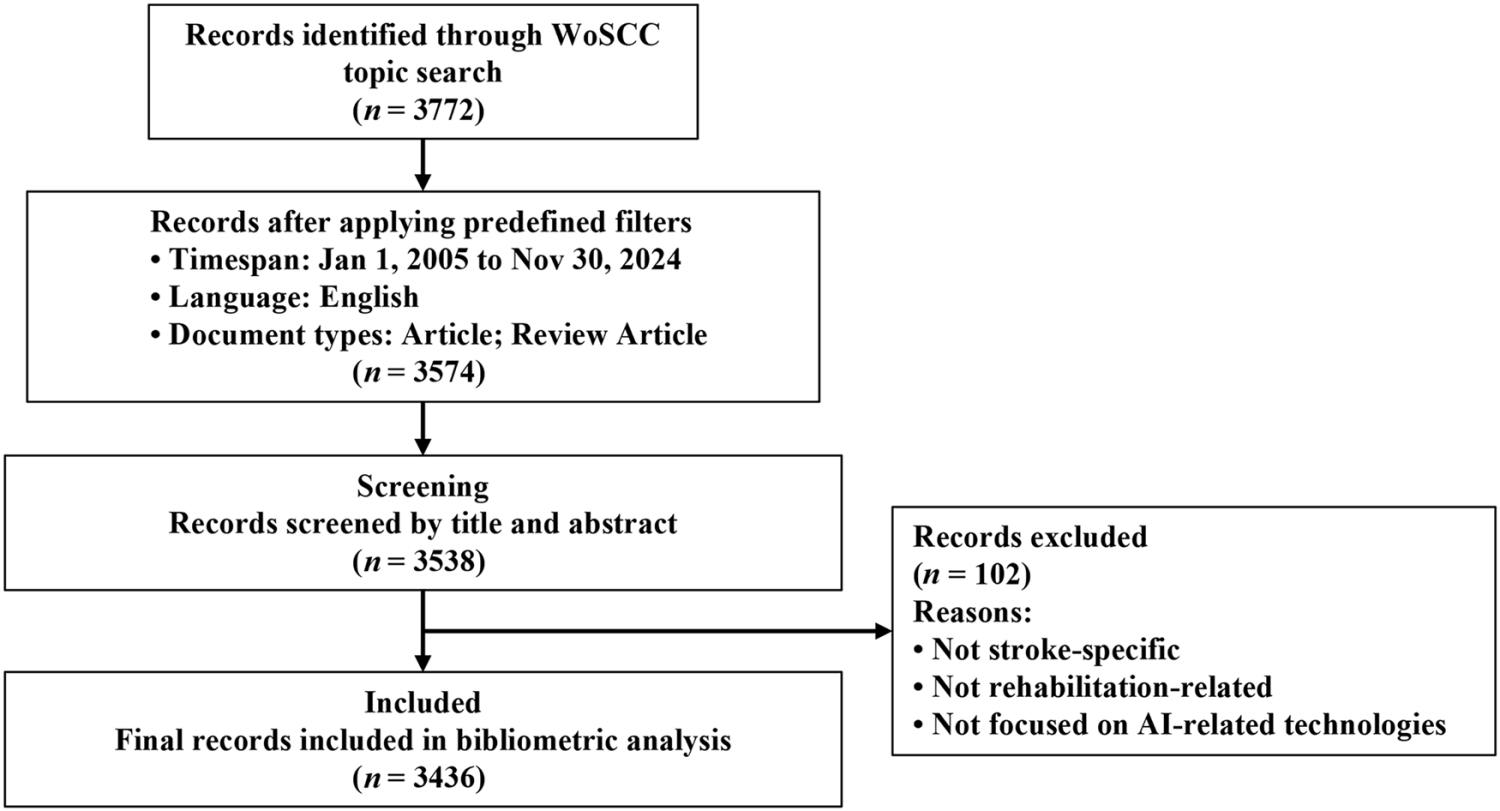
Flow diagram of literature retrieval and screening.

To balance search sensitivity and specificity in this interdisciplinary field, relatively broad topic terms were retained at the initial retrieval stage to capture studies that may have been indexed under heterogeneous terminology. Subsequently, the retrieved records were screened manually based on titles and abstracts, and studies not directly related to stroke‐specific rehabilitation or not focused on AI‐related rehabilitation technologies were excluded. The overall literature retrieval and screening workflow is shown in Figure 1, and the simplified search framework is summarized in Table 1.

**TABLE 1:** Simplified search framework used for literature retrieval.

Search number	Concept block	Search terms
#1	Stroke‐related terms	“stroke” OR “cerebral ischemia” OR “apoplexy” OR “cerebrovascular accident”
#2	AI/digital technology‐related terms	“Artificial Intelligence” OR “AI” OR “Machine Learning” OR “Deep Learning” OR “Virtual Reality” OR “Robotics” OR “VR”
#3	Rehabilitation‐related terms	“Rehabilitation” OR “Recovery”
#4	Final search logic	#1 AND #2 AND #3

*Note*: The search was performed in the Web of Science Core Collection using topic search (TS). The complete search strategy, field restrictions, and applied filters are provided in Table S1.

### 2.2 Data Analysis

Bibliometric analysis was conducted using records retrieved from the WoSCC and exported in plain text format with full records and cited references. The exported data were imported into CiteSpace 6.4.R1 Basic for knowledge mapping and network analysis. The time span was set from 2005 to 2024, with 1 year per slice. In each time slice, nodes were selected using the g‐index criterion with *k* = 25. Cosine similarity was used to calculate link strength, and network simplification was performed using pathfinder and pruning sliced networks.

Separate analyses were performed for publication trends, countries/regions, authors, institutions, journals, cited journals, keywords, and keyword clusters. For keyword‐related analyses, terms were extracted from the topic, abstract, author keywords (DE), and Keywords Plus (ID) fields in WoSCC to construct the keyword co‐occurrence network. Keyword clusters were labeled using the log‐likelihood ratio (LLR) algorithm. Cluster quality was evaluated using modularity *Q* and silhouette *S* values, where higher values indicate better structural significance and internal consistency of the identified clusters.

Burst detection analysis was performed in CiteSpace to identify emerging topics and shifts in research focus over time (Goldberg et al. 2002). No manual adjustment of cluster membership was performed after network generation. In the present study, terms were analyzed as indexed in WoSCC, and no additional manual keyword harmonization was applied prior to network construction.

### 2.3 Tools Used

Three software tools were used in this study. CiteSpace 6.4.R1 Basic was used for network construction, co‐occurrence analysis, co‐citation analysis, clustering, timeline visualization, and burst detection. GraphPad Prism 10.1.2 was used to generate publication trend plots and related statistical graphics. In addition, biblioshiny in R was used to visualize Lotka's law and Bradford's Law. These tools were used in a complementary manner to improve the transparency, interpretability, and reproducibility of the bibliometric results.

## 3 Results

### 3.1 Publication Trends Analysis

**FIGURE 2:**
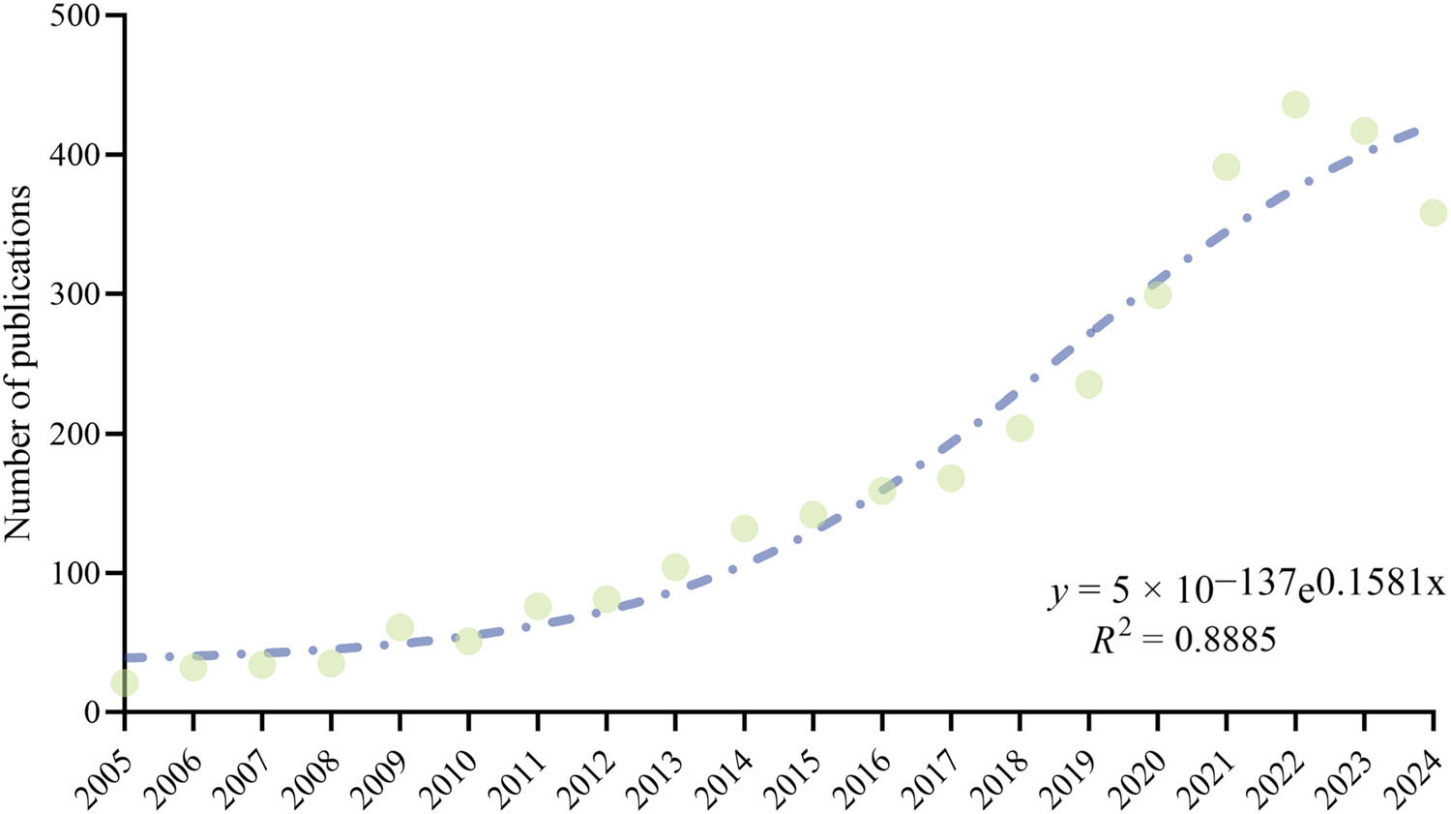
Publication trends of AI in stroke rehabilitation (2005–2024).

From 2005 to 2024, a total of 3436 original articles were identified, comprising 2788 articles (81.1%) and 648 review articles (18.9%). During this period, the volume of publications exhibited an overall upward trend (*R* ^2^ = 0.8885; Figure 2). Specifically, from 2005 to 2016, the number of publications steadily increased each year. From 2017 to 2022, the growth rate accelerated, reaching a historical peak in 2022.

### 3.2 Country/Region Analysis

**FIGURE 3:**
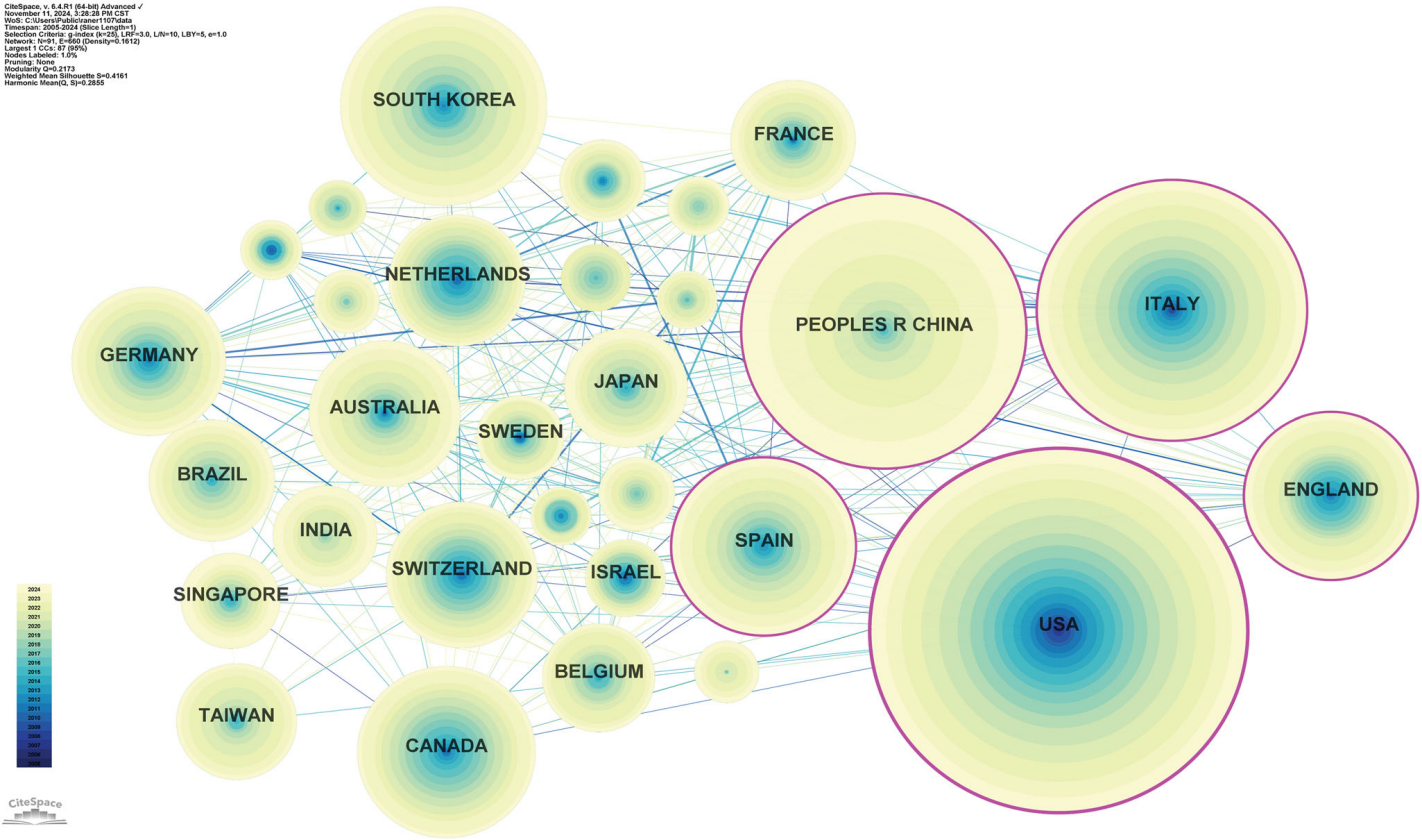
Country/region contribution and collaboration network. Each node represents a country or region, and node size is proportional to the number of publications. Links between nodes indicate collaboration relationships, and the overall network density reflects the degree of international connectivity. Countries with larger nodes contributed more publications to the field, while denser link patterns indicate stronger international collaboration.

A total of 3436 publications on AI in stroke rehabilitation were contributed by researchers from 91 countries/regions. The United States contributed 843 publications, accounting for approximately 24.5% of the total output, followed by China (14.4%) and Italy (12.7%) (Table 2). Other significant contributors include South Korea, Spain, Canada, the United Kingdom, Germany, Switzerland, and Australia. The research network diagram among countries (Figure 3) reveals patterns of collaboration among them (*n* = 91, *E* = 660, density = 0.1612). Network density, an indicator of interconnectedness among nodes, is defined as the ratio of existing edges to potential edges in the network, typically ranging from 0 to 1. A network density of 0.1612 in this study suggests that while some countries have established close collaborations, there is substantial room for enhancement in the connectivity of the global cooperation network.

**TABLE 2:** Top 10 countries/regions contributing to AI‐related stroke rehabilitation research.

Rank	Country/region	Centrality	Publications	Percentage
1	USA	0.45	843	24.5%
2	China	0.12	494	14.4%
3	Italy	0.11	438	12.8%
4	South Korea	0.05	269	7.8%
5	Spain	0.18	210	6.1%
6	Canada	0.06	206	6.0%
7	England	0.11	189	5.5%
8	Germany	0.07	155	4.5%
9	Switzerland	0.02	153	4.5%
10	Australia	0.08	144	4.2%

### 3.3 Author Analysis

**FIGURE 4:**
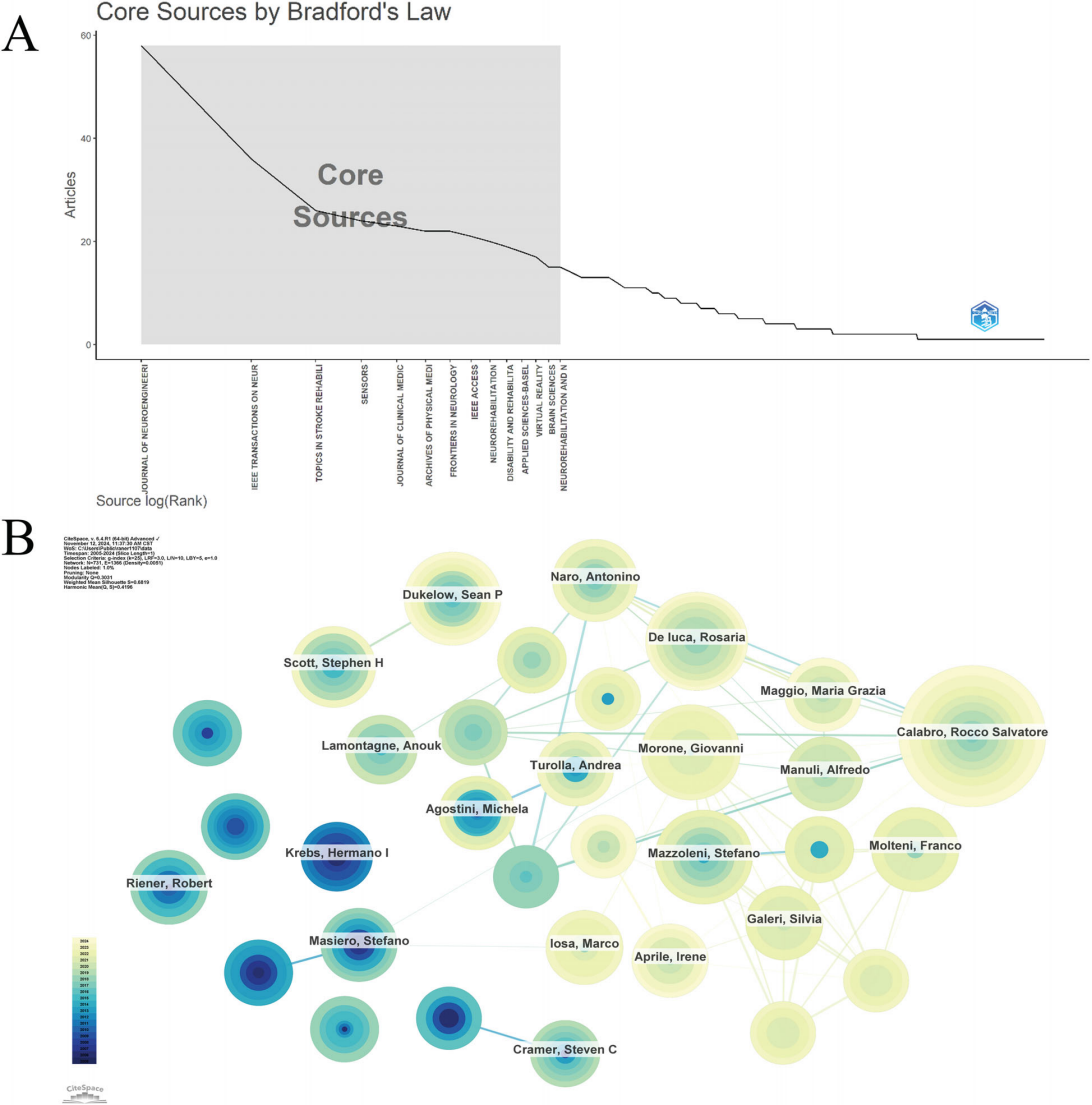
Author analysis. (A) Lotka's law distribution of author productivity. (B) Author collaboration network, in which each node represents an author and node size reflects publication output. Links indicate co‐authorship relationships between authors. A sparse network structure suggests relatively dispersed collaboration patterns across the field.

A total of 731 researchers have contributed to the publication. Author productivity typically adheres to Lotka's law, which posits that a small number of prolific authors produce most of the work, while the majority publish infrequently (Figure 4A). Rocco Salvatore Calabro of the University of Parma was the most productive author among the top 10 authors, with 51 publications and 9090 citations, averaging 178.24 citations per article (Table 3). The author collaboration network (*n* = 731, *E* = 1366, density = 0.0051; Figure 4B) shows a relatively dispersed pattern with few dense collaborative relationships.

**TABLE 3:** Top 10 authors in AI‐related stroke rehabilitation research.

Rank	Author	Country/region	Institution	Publications	Citations	Average citations per paper
1	Calabro, Rocco Salvatore	Italy	University of Parma	51	9090	178.24
2	De luca, Rosaria	Italy	IRCCS Bonino Pulejo	24	2615	108.96
3	Morone, Giovanni	Italy	University of L'Aquila	23	4499	195.61
4	Dukelow, Sean P	Canada	Alberta Health Services (AHS)	23	4225	183.70
5	Mazzoleni, Stefano	Italy	Politecnico di Bari	22	1655	75.23
6	Molteni, Franco	Italy	Valduce Hosp CO	18	4181	232.28
7	Naro, Antonino	Italy	Azienda Osped Univ AOU Policlin G Martino	17	3108	182.82
8	Scott, Stephen H	Canada	Queens University	17	9529	560.53
9	Riener, Robert	Switzerland	University of Zurich	15	12,013	800.87
10	Turolla, Andrea	Italy	University of Bologna	14	3386	241.86

*Note*: Author affiliation and country/region were reported as indexed in WoSCC.

### 3.4 Institution Analysis

**FIGURE 5:**
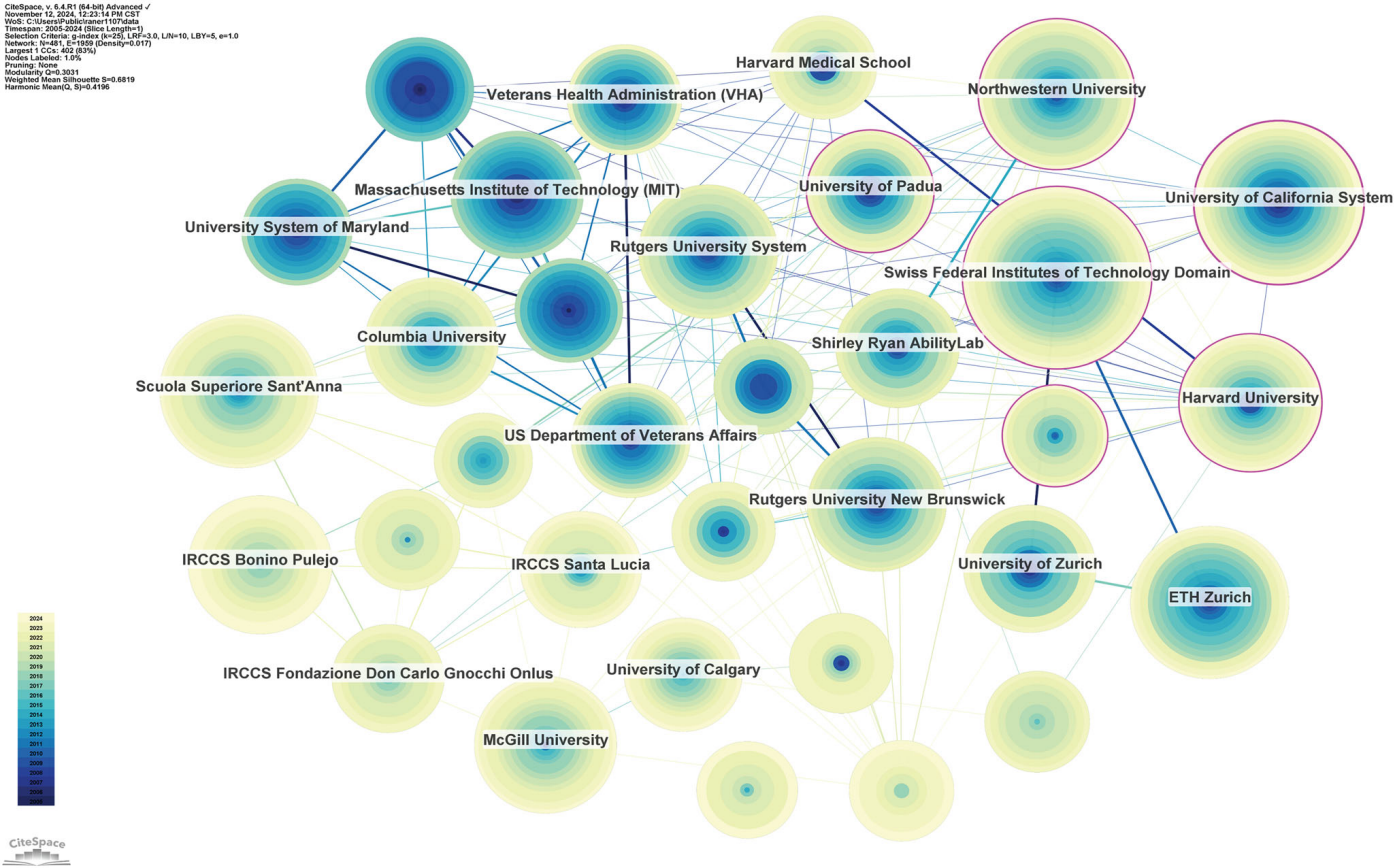
Institutional collaboration network. Each node represents an institution, and node size is proportional to publication count. Links represent collaborative relationships between institutions. The relatively low network density indicates that institutional cooperation in this field remains limited and broadly dispersed.

A total of 481 institutions contributed to the literature. The Swiss Federal Institutes of Technology Domain led with 91 articles, followed by the University of California System publishing 69 articles (Table 4). This indicates the active involvement and research output of these leading institutions in stroke rehabilitation. The institutional research network (*n* = 481, *E* = 1959, density = 0.0017; Figure 5) reveals collaboration patterns among these institutions. The low density suggests limited cooperation, with overall connections being loose and the network broadly dispersed.

**TABLE 4:** Top 10 institutions in AI‐related stroke rehabilitation research.

Rank	Institution	Country/region	Centrality	Publications
1	Swiss Federal Institutes of Technology Domain	Switzerland	0.10	91
2	University of California System	USA	0.20	69
3	ETH Zurich (part of ETH Domain)	Switzerland	0.01	65
4	Scuola Superiore Sant'Anna	Italy	0.08	64
5	Northwestern University	USA	0.13	63
6	Harvard University	USA	0.14	53
7	McGill University	Canada	0.08	51
8	IRCCS Bonino Pulejo	Italy	0.00	51
9	Rutgers University System	USA	0.02	50
10	Rutgers University New Brunswick	USA	0.02	50

*Note*: Institution names were reported as indexed in WoSCC.

### 3.5 Journal Analysis

Research on AI in stroke rehabilitation was published in 1036 journals, with the top 10 journals listed in Table 5. The *Journal of NeuroEngineering and Rehabilitation* ranked first with 238 publications, accounting for 6.93% of all included records, followed by *IEEE Transactions on Neural Systems and Rehabilitation Engineering* (160 publications) and *Frontiers in Neurology* (104 publications). Together, the top 10 journals accounted for nearly 15% of the total publication output.

**TABLE 5:** Top 10 journals publishing AI‐related stroke rehabilitation research.

Rank	Journal	Publications	Percentage	IF
1	*Journal of NeuroEngineering and Rehabilitation*	238	6.93%	5.2
2	*IEEE Transactions on Neural Systems and Rehabilitation Engineering*	160	4.66%	4.8
3	*Frontiers in Neurology*	104	3.03%	2.7
4	*Sensors*	96	2.79%	3.4
5	*Neurorehabilitation*	77	2.24%	1.7
6	*Neurorehabilitation and Neural Repair*	72	2.10%	3.7
7	*Archives of Physical Medicine and Rehabilitation*	69	2.01%	3.6
8	*IEEE Access*	61	1.78%	3.4
9	*Applied Sciences*	58	1.69%	2.5
10	*Topics in Stroke Rehabilitation*	52	1.51%	2.2

*Note*: IF (Impact Factor) statistics are as of November 30, 2024.

**FIGURE 6:**
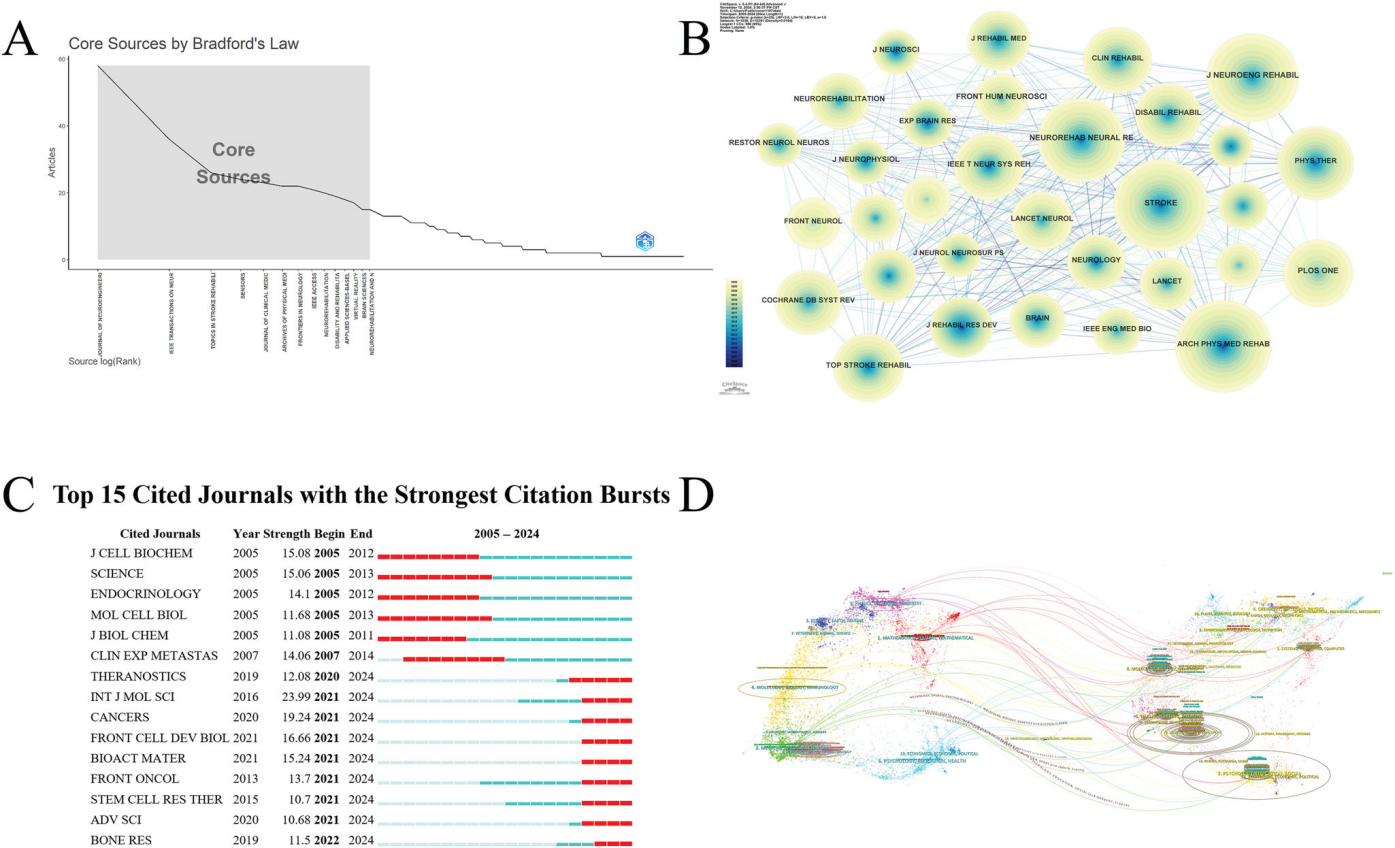
Journal analysis. (A) Bradford's law distribution of journal productivity. (B) Journal collaboration/citation network, where nodes represent journals and links indicate citation‐related relationships. (C) Citation burst analysis of cited journals, showing periods during which specific journals received rapidly increased attention. (D) Dual‐map overlay of journals, illustrating citation flows and disciplinary linkages between citing and cited journals.

Bradford's law analysis further demonstrated an uneven distribution of publications across journals, with a limited number of core journals and a large number of peripheral journals (Figure 6A). The journal collaboration network (*N* = 1036, *E* = 10,391, density = 0.0194) suggested the presence of citation‐related connections among journals, although the overall network remained relatively loose (Figure 6B). Among cited journals, *Stroke* had the highest citation count (2294), indicating that it is widely referenced within the retrieved literature (Table 6). From 2020 to 2024, journals including *Cancers*, *Frontiers in Cell and Developmental Biology*, *Bioactive Materials*, and *Advanced Science* showed marked citation bursts (Figure 6C). The dual‐map overlay further illustrated the citation relationships and disciplinary distributions of the literature included in this study (Figure 6D).

**TABLE 6:** Top 10 cited journals in AI‐related stroke rehabilitation research.

Rank	Cited journals	Citations	Centrality	IF
1	*Stroke*	2294	0.01	7.8
2	*Archives of Physical Medicine and Rehabilitation*	2263	0.01	3.6
3	*Journal of NeuroEngineering and Rehabilitation*	2142	0.03	5.2
4	*Neurorehabilitation and Neural Repair*	1982	0.01	3.7
5	*Physical Therapy*	1478	0.01	3.5
6	*IEEE Transactions on Neural Systems and Rehabilitation Engineering*	1289	0.01	4.8
7	*Clinical Rehabilitation*	1278	0.01	2.6
8	*Topics in Stroke Rehabilitation*	1274	0.01	2.2
9	*PLOS One*	1219	0.02	2.9
10	*Disability and Rehabilitation*	1162	0.01	2.1

*Note*: IF (Impact Factor) statistics are as of November 30, 2024.

### 3.6 Keyword Co‐Occurrence Analysis

A total of 703 distinct keywords were identified across the 3436 included publications. The 10 most frequent keywords are listed in Table 7, with “virtual reality” ranking first (1231 occurrences), followed by “rehabilitation,” “stroke,” “recovery,” and “therapy.”

**TABLE 7:** Top 10 keywords in AI‐related stroke rehabilitation research.

Rank	Keyword	Centrality	Count
1	virtual reality	0.01	1231
2	rehabilitation	0.01	1058
3	stroke	0.01	925
4	recovery	0.01	816
5	therapy	0.03	493
6	upper limb	0.02	431
7	upper extremity	0.03	369
8	stroke rehabilitation	0.03	361
9	reliability	0.03	341
10	motor recovery	0.03	272

**FIGURE 7:**
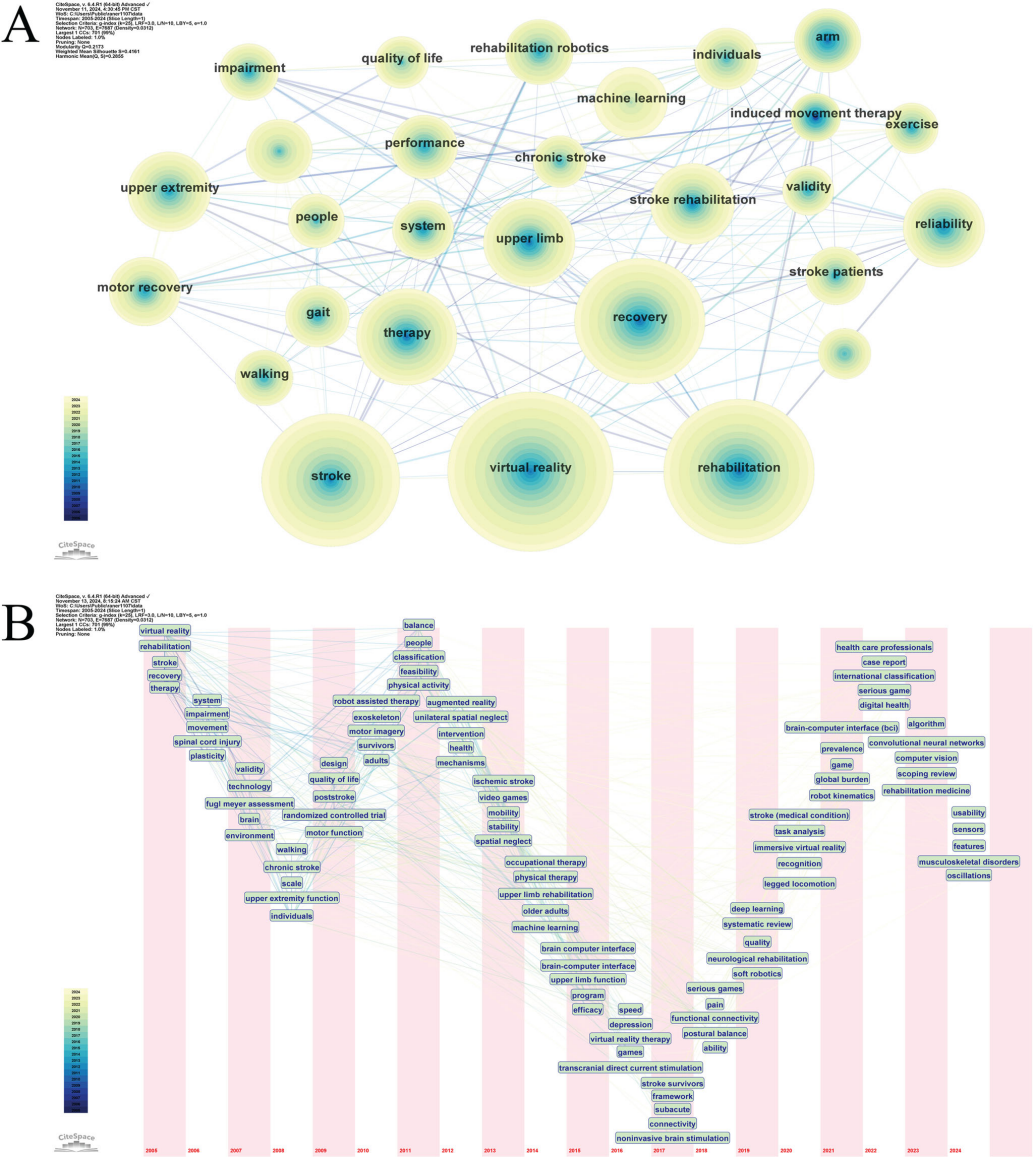
Keyword analysis. (A) Keyword co‐occurrence network constructed from terms extracted from the topic, abstract, author keywords, and Keywords Plus fields of the included records. (B) Keyword time‐zone view showing the temporal evolution of major terms across the study period.

A keyword co‐occurrence network was constructed using terms extracted from the topic, abstract, author keywords, and Keywords Plus fields of the included records (*N* = 703, *E* = 7687, density = 0.0312; Figure 7A). The relatively dense connections among keywords suggest close thematic relationships within the field. The time‐zone view (Figure 7B) further showed the temporal evolution of high‐frequency terms.

### 3.7 Keyword Clustering Analysis

**FIGURE 8:**
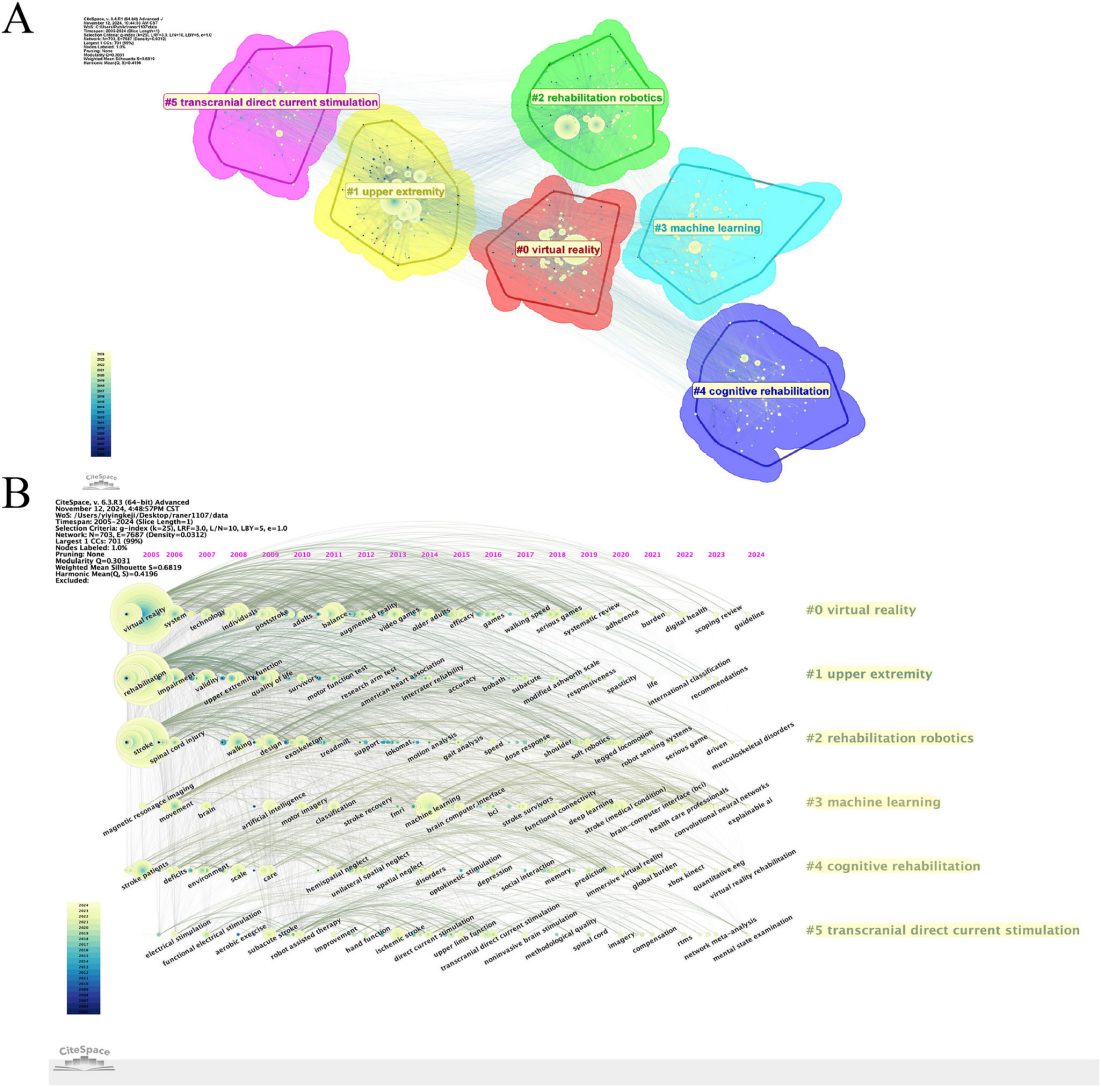
Keyword clustering analysis. (A) Keyword cluster view generated from the keyword co‐occurrence network. Each colored cluster represents a thematic grouping of closely related keywords, and cluster labels were assigned using the log‐likelihood ratio (LLR) algorithm. (B) Timeline view of keyword clusters, showing the temporal evolution and persistence of major thematic clusters across the study period.

Using the LLR algorithm, keyword clustering analysis identified six major thematic clusters within the literature (Figure 8A). The weighted mean silhouette value (*S* = 0.6819) exceeded the commonly accepted threshold of 0.5, indicating reasonable internal consistency, while the modularity value (*Q* = 0.3031) exceeded 0.3, supporting the statistical significance of the overall cluster structure. The six major clusters were labeled as #0 “virtual reality,” #1 “upper extremity,” #2 “rehabilitation robotics,” #3 “machine learning,” #4 “cognitive rehabilitation,” and #5 “transcranial direct current stimulation.” The timeline view (Figure 8B) further suggested that these clusters were not fully independent, but showed partial convergence over time.

### 3.8 Keyword Burst Analysis

**FIGURE 9:**
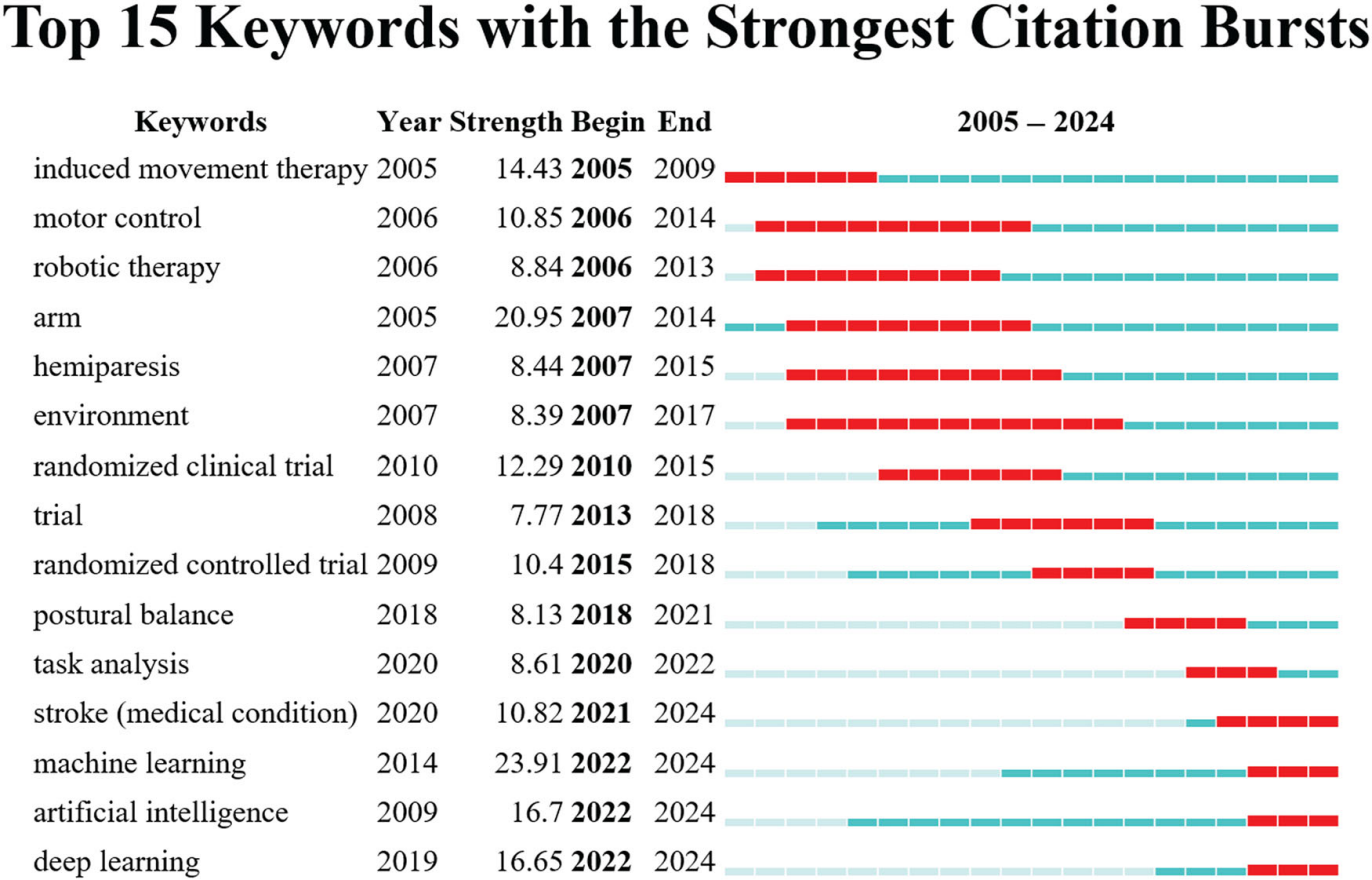
Keyword burst view. The figure shows keywords with the strongest citation bursts over time. Red segments indicate the periods during which a keyword experienced a marked increase in attention, reflecting emerging topics or rapidly expanding research interest.

Burst detection analysis identified 15 keywords with the strongest citation bursts over time (Figure 9). These burst terms reflect periods of rapidly increased research attention and help identify emerging topics within the field. From 2022 to 2024, “machine learning,” “artificial intelligence,” and “deep learning” showed particularly strong citation bursts.

## 4 Discussion

This study provides a comprehensive bibliometric overview of AI‐related research in stroke rehabilitation over the past two decades, showing substantial growth in publication output and clear shifts in thematic emphasis. The field appears to have evolved from earlier hardware‐ and platform‐centered rehabilitation approaches toward more adaptive, data‐driven, and algorithm‐informed directions. Notably, these patterns should be interpreted as changes in research activity and thematic structure, rather than as direct evidence of clinical effectiveness or routine implementation.

### 4.1 Global Research Landscape

The global research landscape of AI in stroke rehabilitation is characterized by marked geographic and institutional concentration. Publication output is dominated by the United States, China, and several European countries, indicating that the field has been driven largely by a limited number of high‐output research systems. At the same time, country‐level collaboration remained only moderately connected, suggesting that international cooperation still has considerable room for strengthening. This concentration in high‐resource settings has important implications for interpretation. The current literature is shaped mainly by countries with stronger research capacity, digital infrastructure, and rehabilitation technology ecosystems, whereas low‐resource and developing regions remain less visible despite often facing substantial rehabilitation needs. This pattern is consistent with prior reviews showing that stroke rehabilitation in low‐ and middle‐income countries is constrained by limited service capacity, workforce shortages, financing barriers, and unequal access to care (Kayola et al. 2023; Bernhardt et al. 2020; Gandhi et al. 2024). It also suggests not only unequal research output but also different conditions for adoption and implementation. Technology‐intensive approaches, such as robotics, VR, and data‐rich monitoring systems, may be more feasible in settings with stronger infrastructure and institutional support, whereas lower‐resource settings may face greater constraints related to affordability, workforce training, digital access, and long‐term rehabilitation delivery (de Andrade et al. 2026; Jarvis et al. 2024; Verma et al. 2022). Accordingly, although AI‐related rehabilitation technologies are often discussed as potential tools for reducing disparities in care, their real‐world uptake is likely to vary across healthcare systems with different levels of economic and institutional support.

At the institutional and author levels, the literature also remains concentrated around a limited number of productive centers and frequently cited researchers, while collaboration networks remain relatively fragmented. This suggests that strong productivity has not yet translated into dense collaborative integration across the field, and that broader cross‐institutional and cross‐national cooperation may still be needed to support more balanced development of AI‐related rehabilitation research.

Journals play an important role in shaping the dissemination of research in this field. A relatively small number of core journals accounted for a substantial proportion of publications, while citation bursts in journals spanning rehabilitation, bioengineering, and materials science suggest increasing multidisciplinary engagement. Citation‐based indicators, however, should be interpreted cautiously. High citation counts may reflect publication age, field size, article type, self‐citation practices, and database indexing patterns, rather than directly indicating greater clinical importance or technological maturity. Accordingly, highly cited journals, authors, or publications should be understood primarily as having greater visibility within the retrieved literature, rather than being assumed to represent the most clinically important or technologically advanced work in the field.

### 4.2 Clinical and Methodological Structure of the Field

Keyword and clustering analyses suggest that the current literature is not evenly distributed across rehabilitation domains, but is instead organized primarily around a motor rehabilitation axis. This pattern is reflected by the dominance of clusters such as “virtual reality,” “upper extremity,” and “rehabilitation robotics,” together with the high‐frequency occurrence of terms including “virtual reality,” “upper limb,” “upper extremity,” and “motor recovery.” Taken together, these findings indicate that upper‐limb recovery and technology‐assisted motor training remain the most visible clinical contexts in AI‐related stroke rehabilitation research. In this setting, VR has been widely studied as an immersive rehabilitation platform associated with patient engagement, task repetition, and adaptive training design, while rehabilitation robotics has been prominently represented as a platform for repetitive and standardized upper‐limb training (Kiyono et al. 2024; Dixit et al. 2024; N. Zhang et al. 2024). By comparison, nonmotor domains appear in the present bibliometric structure as smaller but identifiable directions rather than dominant research axes. Specifically, “cognitive rehabilitation” and “transcranial direct current stimulation” emerged as distinct clusters, suggesting that cognitive and neuromodulation‐related rehabilitation approaches have gained increasing visibility in the recent literature (Albizu et al. 2023; Renati et al. 2021; Reinhardt et al. 2020). Domains such as language rehabilitation, psychosocial reintegration, and community‐based long‐term recovery are not prominently reflected in the current keyword and clustering structure, suggesting that they are less visible in the present AI‐related literature than motor rehabilitation‐related topics.

The clustering and timeline analyses further suggest that AI‐related approaches in this field do not follow a single methodological pathway. Rather, the present bibliometric structure points to at least two broad patterns. One is centered on intervention‐oriented technology platforms, particularly VR and rehabilitation robotics, which are closely associated with motor rehabilitation‐related domains. The other is centered on data‐driven methodological directions, especially machine learning, which appears not only as a standalone thematic cluster but also as a methodological direction that becomes increasingly visible over time alongside cognitive rehabilitation, rehabilitation robotics, and neuromodulation‐related themes. In this sense, the timeline view suggests a gradual shift from predominantly platform‐centered rehabilitation technologies toward a more methodologically layered field, in which algorithmic approaches are increasingly represented across multiple rehabilitation contexts. This interpretation is further supported by representative studies in the recent literature. Machine learning‐related approaches have increasingly been applied to functional prognosis and rehabilitation outcome prediction after stroke, suggesting a growing role for algorithmic methods in assessment‐oriented and prediction‐oriented pathways (Campagnini et al. 2022; Harari et al. 2020; Apostolidis et al. 2024). At the same time, more specialized AI‐related directions, including AI‐assisted aphasia evaluation and treatment, computer vision‐based movement analysis, and brain–computer interface‐related rehabilitation research, indicate increasing methodological diversification beyond the dominant platform‐centered themes (Huber et al. 2024; Silvoni et al. 2011). These findings suggest that AI in stroke rehabilitation may be better understood as a set of domain‐specific and method‐specific trajectories with unequal levels of visibility and translational maturity, rather than as a single homogeneous field.

### 4.3 Interpretive Insights and Future Directions

The burst keyword analysis provides additional insight into the temporal evolution of the field. Early burst terms, such as induced movement therapy, motor control, robotic therapy, arm, and hemiparesis, indicate that the initial development of AI‐related stroke rehabilitation research was closely tied to motor recovery and technology‐assisted physical training. In the intermediate phase, burst terms including randomized clinical trial, trial, randomized controlled trial, postural balance, and task analysis suggest increasing attention to clinical evaluation, intervention testing, and more refined functional assessment. By contrast, the most recent burst terms, particularly machine learning, AI, and deep learning, indicate that the field has increasingly shifted toward algorithm‐driven and data‐oriented research directions.

This temporal pattern suggests that the evolution of the field is not characterized by simple replacement of one dominant technology by another, but rather by a gradual broadening of methodological emphasis. Early research was primarily organized around intervention platforms for motor rehabilitation, whereas more recent work increasingly incorporates computational models for prediction, assessment, and individualized rehabilitation planning (Zu et al. 2023). At the same time, representative studies on AI‐assisted aphasia evaluation and treatment, computer vision‐based movement analysis, and brain–computer interface‐related rehabilitation research further suggest that the methodological profile of the field is becoming more diverse (Zhong 2024).

From a poststroke rehabilitation perspective, these temporal shifts suggest several clinically relevant priorities for future research. Although the most visible literature remains concentrated in motor rehabilitation and technology‐assisted training, future progress will likely require broader extension of AI‐related methods into other clinically relevant domains, such as cognitive recovery, aphasia‐related rehabilitation, psychosocial reintegration, and community‐based long‐term management. In addition, the increasing visibility of algorithm‐driven approaches suggests that future studies should place greater emphasis on prognosis‐informed stratification, multimodal functional assessment, and individualized rehabilitation planning. Ultimately, the future clinical value of AI in stroke rehabilitation is likely to depend not only on methodological innovation but also on whether these approaches can be translated into interpretable, scalable, and context‐sensitive rehabilitation pathways across diverse care settings.

### 4.4 Strengths and Limitations

The present study offers interpretive value beyond a descriptive summary of publication trends. First, the bibliometric structure of the field suggests that current AI‐related stroke rehabilitation research is organized primarily around a motor rehabilitation axis, with upper‐limb recovery, VR, and rehabilitation robotics forming the most visible thematic core. Second, the study shows that AI in stroke rehabilitation is not represented as a single uniform methodological category, but rather as a heterogeneous combination of intervention‐oriented platforms and increasingly visible data‐driven approaches, particularly machine learning. Third, by integrating publication patterns, clustering structure, and temporal evolution, the study highlights uneven visibility across rehabilitation domains and across global research settings, suggesting that methodological expansion has not necessarily been matched by balanced domain coverage or translational reach. Taken together, these findings provide a more clinically grounded and structurally differentiated understanding of the field.

Several limitations should be acknowledged. First, the analysis was based solely on the WoSCC, which may underrepresent engineering‐oriented publications, conference proceedings, and computer science studies indexed more comprehensively in other databases. Second, only English‐language articles and review articles were included, which may have introduced language and document‐type bias. Third, keyword‐based retrieval and clustering are affected by heterogeneous terminology and indexing practices across AI, digital health, and rehabilitation research, which may lead to imperfect classification of some AI‐related technologies. Fourth, bibliometric analysis can map research activity and thematic evolution, but it cannot directly assess study quality, clinical effectiveness, or translational readiness. In addition, citation‐based indicators are shaped by publication age, article type, self‐citation, and database coverage, and thus should be interpreted as indicators of visibility rather than direct measures of scientific or clinical importance. Finally, because AI technologies are evolving rapidly, the bibliometric landscape described here may change over time. Moreover, the observed growth of publications may partly reflect broader enthusiasm for AI‐related research, rather than fully validated clinical translation across stroke rehabilitation settings.

## 5 Conclusion

This study provides a comprehensive bibliometric overview of AI‐related research in stroke rehabilitation from 2005 to 2024. The findings demonstrate sustained growth in publication output and suggest a thematic shift toward technology‐assisted, data‐driven, and adaptive rehabilitation approaches. Motor rehabilitation‐related domains, particularly VR, rehabilitation robotics, and machine learning‐supported research, remain the most visible themes in the literature, while cognitive rehabilitation, neuromodulation‐related topics, and other emerging AI‐oriented directions are receiving increasing attention. The bibliometric landscape also reveals important heterogeneity across clinical rehabilitation domains, AI methodological subfields, and global research participation. The value of this study lies not only in mapping publication trends but also in identifying underexplored rehabilitation domains, differentiating AI‐related methodological pathways, and highlighting translational and collaborative gaps across the field. These findings may help inform future interdisciplinary research and translational exploration, although the clinical effectiveness and implementation readiness of specific technologies require further validation through outcome‐based research.

## Author Contributions

**Yuhua Li**: conceptualization, methodology, validation, writing – review and editing, writing – original draft, **Weixi Liu**: software, data curation, investigation, visualization, formal analysis, writing – original draft, writing – review and editing, validation, **Xiaomei Yuan**: writing – review and editing, validation, project administration, supervision, resources, writing – original draft, visualization

## Funding

The authors have nothing to report.

## Supporting information




**Supplementary Materials**: brb371451‐sup‐0001‐TableS1.docx

## Data Availability

The data that support the findings of this study are openly available in Web of Science Core Collection at https://www.webofscience.com/wos/woscc.
