# Supplementary material for: Artificial Intelligence in Stroke Rehabilitation: A 20‐Year Bibliometric Analysis of Digital Health Trends and Technologies
Source: Brain Behav. 2026 Apr 27;16(5):e71451. doi: 10.1002/brb3.71451 (PMC13118396; doi:10.1002/brb3.71451)
Supplement: Supplementary file 1 — Supplementary Materials: brb371451‐sup‐0001‐TableS1.docx [file BRB3-16-e71451-s001.docx]

Supplementary Table S1. Full search strategy used in the Web of Science Core Collection

| **Item** | **Content** |
| --- | --- |
| Database | Web of Science Core Collection (WoSCC) |
| Indexes | SCI-Expanded; SSCI |
| Search date | November 30, 2024 |
| Search field | TS = Topic Search |
| TS coverage | Title, Abstract, Author Keywords, and Keywords Plus |
| Timespan | January 1, 2005 to November 30, 2024 |
| Language filter | English |
| Document type filter | Article; Review Article |
| Query #1 (stroke-related terms) | TS = ("stroke" OR "cerebral ischemia" OR "apoplexy" OR "cerebrovascular accident") |
| Query #2 (AI/digital technology-related terms) | TS = ("artificial intelligence" OR "AI" OR "machine learning" OR "deep learning" OR "virtual reality" OR "VR" OR "robotics") |
| Query #3 (rehabilitation-related terms) | TS = ("rehabilitation" OR "recovery") |
| Final combined query | #1 AND #2 AND #3 |

Screening procedure: After retrieval, records were screened manually based on titles and abstracts. Studies were excluded if they were not directly related to stroke-specific rehabilitation or were not focused on AI-related rehabilitation technologies. The final dataset included 3,436 publications for bibliometric analysis.
